# Stochastic, structural and functional factors influencing AMPA and NMDA synaptic response variability: a review

**DOI:** 10.1042/NS20160051

**Published:** 2017-06-14

**Authors:** Vito Di Maio, Francesco Ventriglia, Silvia Santillo

**Affiliations:** Istituto di Scienze Applicate e Sistemi Intelligenti del CNR, Via Campi Flegrei 34, 80078, Pozzuoli, Italy

**Keywords:** AMPA, EPSC, LTP, NMDA, synaptic transmission, Synaptic Modeling

## Abstract

Synaptic transmission is the basic mechanism of information transfer between neurons not only in the brain, but along all the nervous system. In this review we will briefly summarize some of the main parameters that produce stochastic variability in the synaptic response. This variability produces different effects on important brain phenomena, like learning and memory, and, alterations of its basic factors can cause brain malfunctioning.

## Introduction

The brain is the most complex information processing “machine”. Not only does it process and produce responses to all the inputs coming from the environment, it also stores information (memory) and elaborates it by a precise control of synaptic strength (synaptic plasticity), and performs high-level cognitive tasks. Neurons are the building blocks of the brain, and their mutual connections (synapses) are the basic elements for the transfer of information between different areas of the brain.

While neurons are the basic elements of the brain's functional neural networks, synapses, being the effective determinants of the neuronal output (neural code formation), can be considered the computational machinery of the individual neuron. Moreover, alterations in the synaptic functionality produce severe modifications in the way the neurons, and hence the brain, process information and store memory [1].

Most synapses in the brain are of the excitatory type and almost 90% of these use glutamate as neurotransmitter (glutamatergic synapses). It is therefore not surprising that a huge amount of research projects and data in the literature, both experimental and theoretical, relate to their study.

Glutamatergic post-synaptic response is mediated by two types of co-localized, ionotropic, receptors: the α-amino-3-hydroxy-5-methyl-4-isoxazole propionic acid sensitive receptors (AMPARs) and the *N*-methyl-d-aspartate sensitive receptors (NMDARs) ([2], among many others). Both receptor types, if bound to glutamate molecules, produce a depolarizing current known as an excitatory post-synaptic current (EPSC), which induces an excitatory post-synaptic potential (EPSP). AMPARs and NMDARs have different dynamics and roles thatcontribute differently in shaping the EPSC (and thus the EPSP) ([3-5], among many others).

Glutamatergic synaptic transmission is quite complex and a full description of all the mechanisms involved in its regulation is beyond the scope of this review. Herein, we will only describe some of the most important factors that determine the variability of the synaptic response. We will focus mainly on some models, and some solutions, that we have proposed in the past decade of work in the attempt to understand the role of the basic parameters that shape synaptic responses, since not all can be fully determined experimentally.

**Figure 1:**
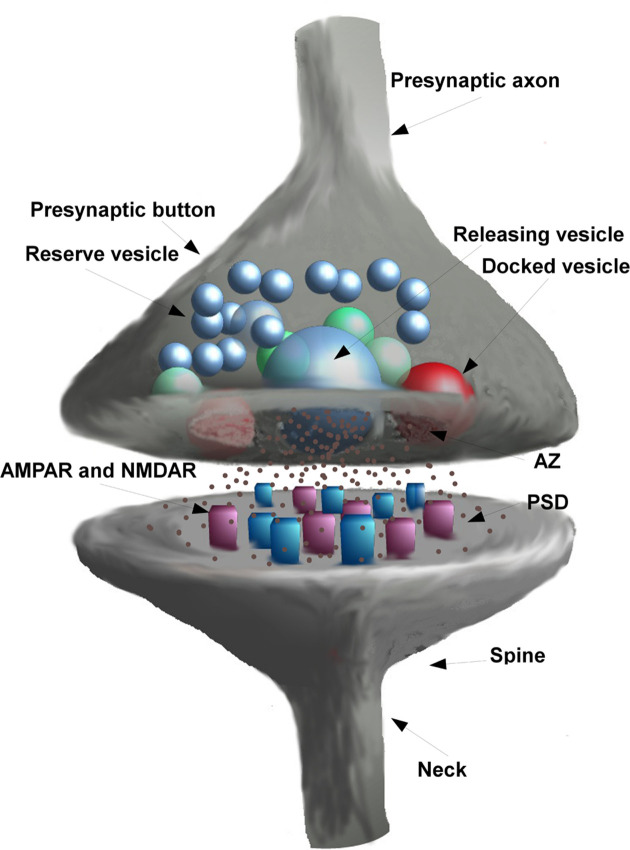
The glutamatergic synaptic structure.

After a brief and simple explanation of the synaptic structure and functionality (see the very simplified diagram of the synaptic structure presented in Figure 1), the review will focus on: the synaptic models (the geometry of the synaptic space, diffusion model of glutamate, model of the post-synaptic response) while discussing pre-, intra- and post-synaptic factors.

As shown in Figure 1, the pre-synaptic end [active zone (AZ)] contains vesicles, some of which are docked to the cell membrane and one of which releases glutamate in the synaptic cleft following a pre-synaptic spike. On the post-synaptic end [post-synaptic density (PSD)], receptors can be activated if bounded by glutamate.

## Synaptic models

### Geometry of the synaptic space

**Figure 2:**
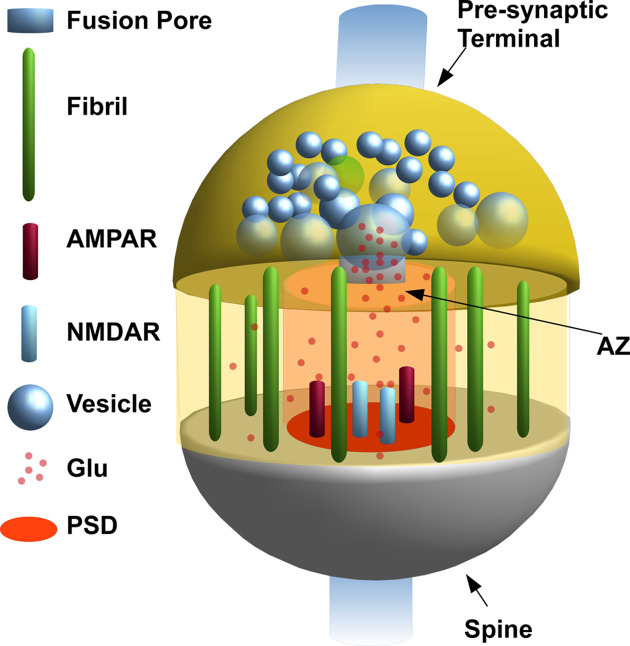
Geometric model of the synaptic space.

Although the structure of the glutaminergic synapse appears to be simple in principle, its geometry and the diffusion of transmitters have several infrastructure limitations that need to be accounted for in a correct simulation [6-13]. Several models oversimplify the synaptic space, and the diffusion mechanism, just assuming the instantaneous release of a given amount of glutamate molecules at the top of a parallelpiped, and an array of receptors at its bottom [14,15]. Other models consider a much complex structure of the synaptic space (see for example [5,16]). Our recent version of the synaptic model has a much more detailed and complex geometry that considers three main compartments for diffusion (vesicle, fusion pore and synaptic cleft), the 3D spaces occupied by receptors in the cleft and also the presence of fibrils [4,9,12,13,17,18,19]. A schematic representation of our synaptic space model is shown in in Figure 2. In short, we divide the synaptic cleft in two areas delimited by two concentric cylinders. The inner cylinder is based on the PSD (red circle of Figure 2) and is delimited on top by a circle of the same diameter (AZ). The diameter of this cylinder can vary among synapses since it depends on the number of receptors on the PSD and, consequently, on the synaptic activity and long-term potentiation [20]. Arellano et al [21] have estimated a range between 0.01 and 0.33 μm^2^ (average value of 0.08±0.06 μm^2^) for the area of the PSD. Schikorski and Stevens [22,23] have found values rather different with an average area of 0.4 μm^2^. In our simulation, we have used a mean diameter of the inner cylinder of 220 nm [4,12,13,17,19,24]. The outer cylinder delimits all the synaptic space and corresponds to the maximum width of the spine head. Since diameter of the spine head can be up to 1 μm [14], we choose a conservative value of 400 nm.

The space between the inner and outer cylinder is filled with fibrils (see Figure 2) that connect pre- and post-synaptic membrane cell [9,25,26]. Fibrils are neuroligin–neurexin complexes and influence the free diffusion of glutamate molecules in the cleft [9]. Their role seems to be fundamental in synaptic development and maturation ([27], and references therein) and the disruption of their arrangement seems to be connected with autism and other mind-associated conditions [1,28]. Fibrils are modelled as thin cylinders with a diameter of 14 nm (see Figure 1 in [9] and references reported therein) and seperated from each other by 6–22 nm.

Release of glutamate occurs when a vesicle, docked at any point on the AZ domain, fuses with the pre-synaptic membrane. The position (*x,y*) on the plane plays an important role in the shaping the time course and amplitude of the post-synaptic response [11,29]. The vesicle is assumed of spheric shape with an inner mean diameter of 12 nm and filled with different numbers of molecules depending on the concentration of glutamate [10,11,14,22,23,30,31].

When a pre-synaptic spike arrives to the AZ domain, the influx of Ca^2+^ activates the synaptic vesicle fusion machinery which basically constists of a complex of proteins (the SNARE complex) ([32] among many others). This machinery produces a fusion pore, which expands with a radial velocity ([18] and see tables in [31], for example). Glutamate molecules can start diffusion into the cleft when the size of the pore become equal to or greater than a molecule of glutamate. We have modelled the pore as a cylinder with a height of 12 nm because of the the thickness of the cell and vesicle membranes (6 + 6 nm).

On the PSD (red circle in Figure 2) the co-localized AMPARs and NMDARs are also modelled as cylinders protruding of 7 nm into the cleft. At the top of each of these cylinders, two circular hot spots, corresponding to the binding sites for glutamate are positioned. So far, the position of the receptors hot spots, is 13 nm from the AZ surface [12,13,19]. The height of both cylinders has been always considered as 20 nm (height of synaptic cleft). To contribute to the synaptic conductance, each receptor needs to be bound by at least two molecules of glutamate. The probability of transitioning to the open state with a single glutamate molecule bound is so low that we did not include this possibility in our model.

Glutamate molecules, in all the synaptic spaces, follow Brownian motion, limited only by the synaptic structures described above. The collision of a molecule with any of the above structures produces its bouncing motion, with the only exceptions being when a molecule hits a hot spot and the lateral wall. In the former case (binding site), the molecule is likely to bind (see below), while for the latter, it is lost from the synaptic space (absorbing boundary). In fact, the glial cells surrounding the synapse, with their high density of glutamate transporters, recover the molecules that have crossed the boundary and so we consider the probability of one of them returning to be negligible.

### Diffusion model of glutamate

Several different methods, for example Monte Carlo simulation (see [33,34], among many others) or similar alternatives (see [7,8,15,33], among many others) are used to simulate molecular Brownian motion. Most diffusion models, however, require both time and space discretization for the numerical simulation. For our diffusion process, we use Langevin equations, and, for numerical simulation, we discretize the time with a very small time step [40×10^−15^ *s* (40 femtoseconds)], but not the space. This method, in addition to the fine geometrical representation of the synaptic space, permits a very fine description of the 3D molecular motion [3,4,10–13,17,19,24,31,35]. In their standard form, Langevin equations are expressed as

1 $$\frac{d}{dt}r_{i}(t)=v_{i}(t)$$

2 $$m\frac{d}{dt}v_{i}=-\gamma v_{i}(t)+\sqrt{2\varepsilon \gamma }\Lambda (t)$$

where **r***_i_*(*t*) is the position vector (*x_i_*,*y_i_*,*z_i_*) of the *i^th^* molecule at time *t* and **v***_i_*(*t*) is its velocity in the 3D space, *m* is the molecular mass, a friction parameter depending on the absolute temperature [$\gamma =k_{B}\frac{T}{D}$, *k_B_* being the Boltzman constant, *D* the diffusion coefficient of glutamate, and *T* the temperature in °*K*] and ε = *k_B_T*. As stochastic force, we have used a Gaussian white noise [$\left\langle \Lambda_{i}(t)\Lambda_{j}(t+\Delta )\right\rangle =\delta_{i,j}\delta (\Delta )$ ] with intensity 2*εγ*.For numerical simulation, the following time discretized equations, have been used

3 $$r_{i}(t+\Delta )=r_{i}(t)+v_{i}(t)\Delta$$

4 $$v_{i}(t+\Delta )=v_{i}(t)-\gamma \frac{v_{i}}{m\text{Δ}}+\frac{\sqrt{2\varepsilon \gamma \Delta }}{m}{\text{Ω}}_{i}$$

where** Ω***_i_* is a random vector with three Gaussian components (*x_i_*,*y_i_*,*z_i_*) with mean 0 and σ = 1.

At time *t*_0_ = 0, all glutamate molecules, positioned inside the vesicle, move with a starting velocity (**v***_i_*), chosen according to a Maxwell distribution. At the same time *t* = 0, the fusion pore start its opening following its radial velocity. For any time *t* > 0 the pore size increases and, when its diameter is equal to the diameter of a glutamine molecule, diffusion into the cleft can start. Molecules are considered massless (virtual points defined only by their *x,y,z* co-ordinates) except when they approach a receptor-binding site (see below).

Glutamate molecules diffusing in the cleft can reach the PSD represented by a 10 × 10 square matrix (**R**: *i*,*j*∈***R***). Each position *i*,*j* ∈ **R** may or may not contain a receptor (1 for AMPAR, 2 for NMDAR and 0 for empty position). The corners of the square matrix, never contain receptors to respect the circular shape of the PSD (see for example Figure 2 in [10]). The relative AMPAR and NMDAR position *i*,*j* ∈ **R** is always chosen randomly.

If a molecule hits the hot spot of a receptor, the probability of it binding is denoted by *P_B_*. In our early papers [10,11,19,31,35] *P_B_* was computed by classical systems ([14,36,37], among many others) based mainly on an induced equilibrium condition for glutamate. However, as we have demonstrated in [35], the equilibrium condition is never achieved during a synaptic event (see Figure 3 in [10] and Figure 1 in [35]). For this reason, and because computing *P_B_* by the classical mass equations is meaningless by using a 40*-*fs time step, we prefer a new method based on geometrical considerations [3,4,9,12,13,17,24]. Our computation of *P_B_* considers the following assumptions: (a) the shape of a glutamate molecule can be approximated to an ellipsoid; (b) glutamate can bind to receptors only from its *γ*-carboxil group (one of sides of the ellipsoid); (c) the receptor hot spot for glutamate can be approximated to a circular hole. We argued that all the orientations useful for the binding process can be enclosed in a cone angle and hence *P_B_* can be computed as the ratio between this cone angle and the sphere containing all the possible orientations (see Figure 3 in [12]).

**Figure 3:**
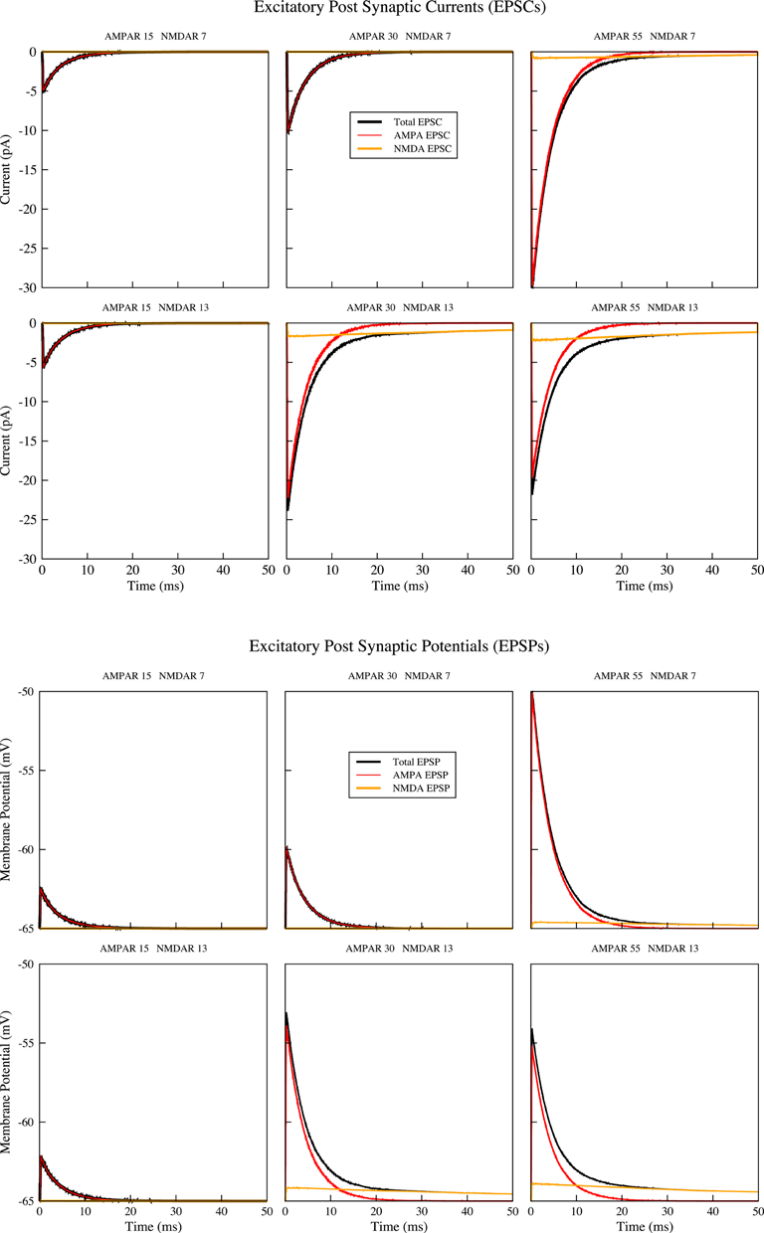
Examples of EPSCs and EPSPs for different number of AMPARs and NMDARs

Because of the small time step and of the fine geometrical description of the synaptic space, the simulation process is computationally very expensive so that to obtain a 500-μs simulation, time we need almost 1 week of computation by using a parallel Fortran program on a parallel cluster of workstations. The final output of the diffusion simulation consists of two 10 × 10 matrices (**B**_1_ and **B**_2_) containing respectively the binding time of first and second glutamate molecule to the receptors, respectively. The matrices **R** and **B**_2_ are used off line by a *C*^++^ program to compute the post-synaptic response. The matrix **B**_1_ is saved as a control since the probability of receptors bound with a single glutamate molecule contributing to the post-synaptic response is negligible (see next section).

### Model of the post-synaptic response

AMPARs and NMDARs, once bound by glutamate molecules, are likely to open their ionic channel permitting the flow of a depolarizing ionic current (inward current). The functional activation of the two types of receptors is, however, very different either because of their different affinities for glutamate or because NMDARs are blocked by Mg^2+^. For NMDARs, then, the binding of glutamate is a necessary but not the only condition needed to contribute to the EPSC. Mg^2+^ unblocking depends on its concentration ([Mg^2+^]) and on the level of the membrane potential (*V_m_*). A sigmoid dependence of NMDAR conductance (*g*) on *V_m_* has been described for different Mg^2+^ concentrations [38,39]. In our recent works, we have expressed the relationship conductance/voltage in terms of unblocking probability (*P_u_*)

5 $$P_{u}(X,V_{m})=\frac{1}{1+Xe^{-\kappa V_{m}}}$$

6 $$P_{u}(V_{m}|\left[{\mathrm{Mg}}^{2+}\right])=\frac{1}{1+[{\mathrm{Mg}}^{2+}]e^{0.1V_{m}}}$$

where *X* is the [Mg^2+^] and *k* is a parameter to fit the curves of [38] and [39]. Our results have shown that, the fast current produced by the AMPA conductance activation, is sufficient to depolarize the membrane unblocking some of the NMDARs at physiological concentrations of Mg^2+^ (∼1 mM) [3,4,17].

A matrix **U**, containing the time of unbinding $\left(t_{u_{i,j}}\in U\right)$ of the second glutamate molecule, was computed by the **B** according to a Poisson stochastic process such that

7 $$t_{u_{i,j}}=t_{b_{i,j}}+P({\bar{\alpha }}_{r})$$

being $t_{b_{i,j}}\in B$ the binding time of the second molecule to the *i,j* receptor and ${\bar{\alpha }}_{r}$ the mean binding duration for the specific receptor type (AMPAR or NMDAR) [4,17]. The parameter α is considerably different between two receptor types because of their different affinities for glutamate, which is higher for NMDARs than for AMPARs. This difference causes the fast decay of the AMPA-dependent response (less then 10 ms) and the slow decay of the NMDA one (up to 500 ms) [36].

Although more complex glutamate-receptor binding dynamics can be used ([40], for example) we adopted the following simplified schema:

for AMPARs

$$R_{0}⇌R_{1}⇌R_{2}⇌R_{2_{o}}⇌R_{1}$$

and for NMDARs

$$R_{0}^{*}⇌R_{1}^{*}⇌R_{2}^{*}⇌R_{2}⇌R_{2_{o}}⇌R_{1}$$

The subscript *“o”* indicates *“open”*, the superscript “*” represents the *“Mg^2+^-block”*, subscripts 0, 1 and 2 indicate not bound, single bound and double bound, respectively.

For the computation of the post-synaptic response, the transition $R_{2}⇌R_{2_{o}}$ is the important one because only receptors in $R_{2_{o}}$ contribute to the synaptic conductance. During the binding time, the transition $R_{2}⇌R_{2_{o}}$ depends on the open probability (*P_o_*) which is different for AMPARs and NMDARs (see [2] and, for numerical values, Table 2 in [4]). To determine the open/close state of each receptor at any time we have used the Heaviside function and the Uniform distribution (*U*(0,1)) such that

$$P_{R_{2}-R_{2_{o}}}=\{\begin{array}{l} 1\;if\;P_{o}\leq U(0,1)\\ 0\;if\;P_{o}>U(0,1) \end{array}P_{R_{2_{o}}-R_{2}}=\{\begin{array}{l} 1\;if\;P_{o}>U(0,1)\\ 0\;if\;P_{o}\leq U(0,1) \end{array}$$

All the receptors in $R_{2_{o}}$ at a given time *t*, contribute to the total synaptic conductance (*g_s_*(*t*)) with its own conductance *g_i,j_*.

The value *g_i,j_* is another important parameter because it differs not only between AMPARs and NMDARs but also within the same receptor type, depending on its subunit composition. In fact, AMPARs and NMDARs are a dimer of dimers, and the dimers composition determines their single channel conductance [41-45]. To have a good representation of all AMPARs and NMDARs conductances and to generalize the model, we have used a Gaussian function $\left(G({\bar{g}}_{r},\sigma_{gr}\right)$ with ${\bar{g}}_{r}$ being the mean conductance for the different dimer compositions and σ*_gr_* the related standard deviation [3,4,17,24,41,42,43,45]. So far, at any time *t* the total conductance for our 10 × 10 receptor matrix will be given by

8 $$g_{s}(t)=\sum_{i=0}^{i=10}\sum_{j=0}^{j=10}g_{r_{i,j}}(t)$$

where $g_{r_{i,j}}(t)$ is the conductance at the time *t* of the receptor *r_i,j_* ∈ **R** and so

$$g_{r_{i,j}}(t)=\left\{ \begin{array}{l} g_{AMPA}\;if\;r_{i,j}\in R\;is\;an\;AMPA\;in\;R_{2_{o}}\\ g_{NMDA}\;if\;r_{i,j}\in R\;is\;an\;NMDA\;in\;R_{2_{o}}\\ 0\;in\;any\;other\;condition \end{array}\right.$$

The EPSC (synaptic current) is then computed as

9 $$I_{s}(t)=g_{s}(t)(V_{m}(t)-V_{e})$$

where *V_m_*(*t*) is the membrane potential and *V_e_* is the reverse potential (or equilibrium potential) depending on ion concentration and computed according to the Nernst equation.

An important characteristic of the glutamatergic synapse is that the PSD is located on the head of a dendritic spine (see Figure 2). The spine can be considered as a particular electric compartment with a peculiar resistance which somehow make it different from the dendritic shaft [3,4,17,24,46-50]. The spine electrical resistance can vary from few MΩ*s* to the order of GΩ*s* depending on its morphology [46-49,51,52].

Since resistance is an important parameter for the modulation of the EPSC, we define a spine resistance *R_s_* to compute the value of *V_m_* [3,4,17,24]

10 $$V_{m}(t)=R_{s}I_{s}(t)$$

where *V_m_* is the potential recorded exactly at the base of the spine. The value of *R_s_* is fixed for a single simulation and usually we vary it across different computational experiments [4,17]. In [3], we have shown how different values *R_s_*, affecting the value of *V_m_*, can produce a different recruitment of NMDA receptors due to the dependence of NMDA unblocking on the membrane potential (see eqn 6) [3,4]. Since, for a given spine resistance, the current producing the variation of *V_m_* is AMPA-dependent, these results show that *R_s_* can be an important parameter for the co-operation between AMPARs and NMDARs in shaping the post-synaptic response [3,4].

Different numbers of the two receptor types and their relative proportion, produces different contributions of the two receptor types to the amplitude and time-course of EPSC [4]. Figure 3 shows an example of the EPSP and EPSC time course for a resistance of 500 MΩ and for two different combinations of the receptor numbers.

## Discussion

Glutamatergic synapses are the most important system of information transfer and elaboration in the brain. Their massive inputs in almost all the neurons of the brain are the main determinants of the neuronal spike sequences generation (neural code). The variability of the spike sequences for a given input depends, in fact, on the large degree of variability of these synaptic inputs. Several pre-, intra- and post-synaptic factors are at the origin of the synaptic response variability.

We used our model of synaptic transmission to try to explain and/or give interpretations of the basic functionality of the glutamatergic synapse, with a view to contributing to the understanding of brain functionality.

### Pre-synaptic factors

The first pre-synaptic event in synaptic transmission is the release of a vesicle and is described as "quantal". This definition was previously given by del Castillo and Katz [53] for the end plate potential in the frog neuromuscular junction, based on the belief that each single vesicle, more or less, contains the same amount of neurotransmitter. Nowadays, the definition of “quantal release” is more properly applied to packets of neurotransmitter stored in vesicles and, more specifically, to the release of a single vesicle (quantum). The release of a quantum produces a range of 5 to more than 100 pA for the EPSC peak recorded at soma, suggesting that the non-uniformity of quanta is a source of this variability (see, for example, [30,54-58], among many others). Variability among quanta is due to the different size of vesicles [59,60], to their glutamate concentration [30,36,58,59] and to the position of the vesicle on the AZ domain [11,31].

Considering that, vesicle concentration ranges from 60 to 210 mM [14], the size of the vesicle determines the number of glutamate molecules released during the single synaptic event [10,11,14,31,35,36,58] and, consequently, the glutamate concentration time-course in the synaptic cleft [14,31,35].

Different numbers of molecules released by a quantum, directly influence the number of post-synaptic receptors that can contribute to the EPSC. Usually, there are almost 200 vesicles in a synaptic terminal, about 10 of which are docked in different positions of AZ area [22,23]. It follows that different combinations “size/concentration/position” produce post-synaptic response variability of stochastic origin [10,19,31,35].

Moreover, the probability of release of a vesicle following a pre-synaptic spike is, usually, less than 1 and differs among brain areas (see for example [61], among many others). Vesicle release probability is another pre-synaptic stochastic factor affecting information transfer in the brain.

In general, we can say that the most important pre-synaptic factors influencing the variability of the post-synaptic response are *stochastic* in nature.

### Intra-synaptic factors

Some intra-synaptic factors can influence the variability of both the amplitude and the time course of the EPSC [9]. Most of these factors essentially produce variable influences on the free diffusion of glutamate [62] by changing the diffusion coefficient (see definition of *γ* in eqn 2).

The presence of fibrils connecting pre- and post-synaptic cells [26], their position and size [9], for example, can make a significant contribution to shaping the post-synaptic response.

The free glutamate diffusion is also affected by the receptors which, based on PSD, protrude into the synaptic cleft by almost 7 nm, i.e. almost one-third of the cleft's height (20 nm) [12,19,35]. So far, the different number of receptors, not only contributes to the post-synaptic source of variability (see subsequent section: ‘Post-synaptic factors’) but also to the intra-synaptic factors because they affect the synaptic geometry and the Brownian diffusion of glutamate.

In our recent paper [3] we have surmised that glial cell,s by regulating Mg^2+^ concentration, can influence the NMDA contribution to the total EPSC. If, our hypothesis were to be confirmed, this form of regulation could be considered another intra-synaptic contributor to the EPSC variability [3]. Because of their nature, we can define the intersynaptic factors influencing the EPSC as *structural*.

### Post-synaptic factors

The number of receptors on the PSD and their relative proportion, are the most important post-synaptic factors influencing the EPSC variability [3,4,17]. The total number, in fact, has great influence on the EPSC amplitude [3] while the relative proportion (AMPA:NMDA) influences mainly the time course [4].

The synaptic activity (pre-synaptic input frequency) and synaptic maturation regulate the number of receptors on the PSD. More precisely, the synaptic activity, by appropriate input frequencies, can induce long-term potentiation (LTP) and long-term depression (LTD) (adding or removal of AMPARs, respectively) contributing to memory and learning ([4,63-70], among many others).

Post-synaptic variability is also contributed to by the dimeric structures of AMPARs and NMDARs which, being dimer of dimers composed of different subunits, have different conductances depending on the dimeric composition ([2,41]; see also earlier section ‘Model of the post-synaptic response’). Different dimer compositions for receptors can be related to different brain areas, to the level of synaptic maturation and very likely to the receptor's functionality [44,45]. It is very likely that different dimer compositions, also produce variability in the binding probability to glutamate (*P_B_*) and in open probability (*P_o_*).

As general consideration we can say that the post-synaptic factors influencing EPSC variability are very little of stochastic nature and largely of *functional* origin.

## Conclusions

In the present review, we have considered some of the most important factors producing the observed variability of the glutamatergic post-synaptic response. Of course, we have not exhaustively covered the problem. For example, a structural parameter that can have a great influence is spine morphology and its electrical properties, the most important of which is the spine resistance. In our simulations, we have considered *R_s_* as a constant during a single synaptic event by changing it only across the different computational experiments [3]. The spine head can change in size depending on the variation of the receptor number, producing a consequence, a variation of the value of its resistance (*R_s_*). Moreover, spines with different lengths can have different resistance values and this parameter is very likely to vary during a single synaptic event due to osmotic factors induced by Ca^2+^ influx mediated by NMDARs [25,71]. In general, however, the value of the membrane potential (*V_m_*) strongly depend on the *R_s_* (see eqn 10) so that its changes modulate the peak amplitude and/or the time course of the EPSC [3,4,17,24,25,46,71]. Finally, we have also surmised that changes in spine resistance can produce variability and regulation mediated by other neurotransmitters, such as the case of the regulation of a glutamatergic synapse modulated by dopamine in medium size spiny neurons [17,24].

Several other factors can still influence the EPSC variability. The main goal of the present review has been to outline the importance of the study, both experimental and computational, of the synaptic response generation and its variability. Without its full understanding, phenomena like neural code, LTP, LTD, neural network activity and, in general, information processing and computational ability of the brain, may never be fully understood.

## Abbreviations

AMPAR: α-amino-3-hydroxy-5-methyl-4-isoxazole propionic acid sensitive receptor
AZ: active zone
EPSC: excitatory post-synaptic current
EPSP: excitatory post-synaptic potential
LTD: long-term depression
LTP: long-term potentiation
NMDAR: *N*-methyl-d-aspartate sensitive receptor
PSD: post-synaptic density
